# Prediagnostic smoking intensity trajectories and subtype-specific vascular disease risk in a nationwide health screening population

**DOI:** 10.3389/ijph.2026.1610024

**Published:** 2026-10-29

**Authors:** Gyuwon Ra, Haejong Lee, Hyoung-Sun Jeong, Kwang-Soo Lee, Young-Joo Won, Ki-Bong Yoo

**Affiliations:** 1 Health Insurance Research Institute, National Health Insurance Service, Wonju-si, Republic of Korea; 2 Department of Health Administration, Yonsei University - Mirae Campus, Wonju-si, Republic of Korea

**Keywords:** cardiovascular disease, cerebrovascular disease, group-based trajectory modeling, health screening, smoking trajectories

## Abstract

**Objectives:**

We examined associations between prediagnostic smoking trajectories and subtype-specific vascular outcomes.

**Methods:**

Using the National Health Insurance Service Customized Database, we conducted a matched case-control study of 1,106,228 adults in South Korea. The exposure was prediagnostic smoking trajectory group, and the outcomes were cardiovascular and cerebrovascular disease and five subtypes: ischemic heart disease, acute myocardial infarction, cerebrovascular disease, intracranial hemorrhage, and cerebral infarction. Trajectories were identified using group-based trajectory modeling, and conditional logistic regression estimated odds ratios.

**Results:**

Trajectory modeling differentiated never, former, high-decreasing, moderate-stable, and high-stable smoking histories. Overall odds increased with smoking intensity and persistence, from former smoking (OR 1.11) to high-stable smoking (OR 1.97). The high-decreasing group showed lower odds than the moderate-stable and high-stable groups. The strongest associations were observed for acute myocardial infarction (OR 3.51) and cerebral infarction (OR 2.46).

**Conclusion:**

Prediagnostic smoking trajectories revealed a graded vascular risk pattern shaped by intensity, persistence, and reduction, with the greatest excess risks for ischemic outcomes. Repeated smoking histories in screening data may move risk assessment beyond current smoking status and inform earlier cessation intervention.

## Introduction

Smoking remains one of the most important preventable determinants of cardiovascular and cerebrovascular disease. Tobacco use contributes to more than 7 million deaths worldwide each year [1], and smoking-attributable diseases have been estimated to impose approximately US$1.4 trillion in annual economic costs [2]. This burden highlights the need to better characterize smoking histories relevant to vascular disease prevention.

Although the vascular harms of smoking are well established, epidemiologic evidence has relied on smoking status assessed at a single point in time [3–5]. Such assessments have limited ability to distinguish smoking histories that differ in intensity, persistence, and changes over time. Evidence from repeated lifestyle measurements suggests that baseline-only assessments may underestimate associations between average cumulative exposure and major cardiovascular disease risk [6].

Group-based trajectory modeling identifies latent subgroups that share similar longitudinal patterns, providing a framework for summarizing smoking histories over time [7, 8]. Previous trajectory-based studies have linked smoking trajectories to cardiovascular outcomes [9–11], but several have defined trajectories from cohort enrollment, leaving a substantial interval between exposure assessment and disease occurrence. Less is known about smoking patterns in the years immediately before diagnosis, when accumulated exposure and recent changes in smoking intensity may be more relevant to clinical disease onset.

Smoking-related vascular risk may also differ by disease subtype. Cardiovascular and cerebrovascular diseases encompass heterogeneous outcomes, and prior evidence suggests that smoking is not uniformly associated with ischemic and hemorrhagic vascular events [4, 12]. Whether smoking trajectories differ in their associations across vascular disease subtypes has not been well characterized.

For prevention-oriented risk assessment, the key issue is whether longitudinal smoking histories add information beyond a single current-status measure. This question is particularly relevant to screening systems that collect longitudinal behavioral information. In South Korea, the national health examination system repeatedly collects smoking information as part of general health screening for cardiovascular risk prevention and management [13]. Drawing on these longitudinal records, we conducted a nationwide matched case-control study to examine whether smoking trajectories during the 6 years before the index date were associated with overall and subtype-specific cardiovascular and cerebrovascular disease risk.

## Methods

### Data and participants

The data were obtained from the National Health Insurance Service (NHIS) Customized Database, a population-based national healthcare database covering the entire Korean population. The database included eligibility information, health screening and questionnaire data, healthcare utilization claims, and mortality records [14]. Data from 2011 to 2022 were used to assess smoking trajectories during the 6 years preceding the index date and to identify incident cardiovascular and cerebrovascular disease cases from 2017 to 2022. This 6-year preindex period was selected to capture smoking patterns over time and provide sufficient repeated measurements for trajectory modeling. Because NHIS health screening is offered annually or biennially depending on employment type, this period allowed at least three screening opportunities even for biennially screened participants. For cases, the index date was defined as the date of first hospitalization with a principal diagnosis of cardiovascular or cerebrovascular disease.

The study population included NHIS beneficiaries between the ages of 30 and 69 who completed national general health screening from January 2015 through December 2016. In Korea, screening intervals vary by employment type, with annual screening for nonoffice workers and biennial screening for office workers. Participants screened in 2015 and 2016 were pooled to reduce selection bias related to screening year. For individuals screened in both years, the most recent examination was retained, resulting in a baseline population of 18,241,353 individuals. The baseline exclusions included deaths, individuals outside the target age range, medical aid beneficiaries, and individuals with missing covariate data; a total of 471,716 individuals were excluded. Of the baseline population of 17,769,637 individuals, 618,109 potential cases who were first hospitalized with a principal diagnosis of cardiovascular and cerebrovascular diseases between January 1, 2017, and December 31, 2022, were identified. Only individuals with screening records during the 6 years before diagnosis and at least three nonmissing smoking measurements were included. This threshold was applied to provide sufficient longitudinal information for estimating smoking trajectories and classifying participants into trajectory groups [7, 15].

To ensure adequate preindex exposure assessment and exclude prevalent disease, we excluded individuals without a health screening record within 2 years before the index date, as well as those with a history of cancer (ICD-10 codes C00–C99) or cardiovascular and cerebrovascular diseases (ICD-10 codes I20–I25 and I60–I69) during the 6 years preceding the index date. History of disease was defined as at least one hospitalization or three outpatient claims with a principal diagnosis of the corresponding disease, to reduce potential over-ascertainment from miscoding or administrative error. After applying these preindex eligibility criteria, 244,679 cases remained eligible for matching.

The pool of potential controls comprised participants who had no history of cardiovascular and cerebrovascular diseases throughout follow-up. After applying the preindex eligibility criteria, the retained cases were matched to potential controls on sex, age, and insurance premium quantile, an indicator of income-related socioeconomic status. Up to six controls were initially selected for each retained case. Matched controls were then assigned the index date of the corresponding case, after which the same preindex eligibility criteria were applied to matched controls. After excluding incomplete matched sets, the final matched study population comprised 241,163 cases and 865,065 controls (Supplementary Material 1, Supplementary Figure S1).

### Measures

Smoking behavior was assessed using repeated health screening questionnaires and classified as never smoker, former smoker, smoking 1–9 cigarettes per day, smoking 10–19 cigarettes per day, and smoking ≥20 cigarettes per day. This categorization was used to reflect gradations in smoking intensity and maintain comparability with previous studies [9]. Because questions on electronic cigarettes were introduced only in 2018 and were not consistently available over time, only combustible cigarette smoking was included in the trajectory analysis.

The main outcome was cardiovascular and cerebrovascular disease, identified using ICD-10 codes I20–I25 and I60–I69, consistent with definitions used in previous epidemiologic studies [4, 9, 12]. The subtype-specific outcomes included ischemic heart disease (I20–I25), acute myocardial infarction (I21), cerebrovascular disease (I60–I69), intracranial hemorrhage (I60–I62), and cerebral infarction (I63).

Several covariates were included in the analysis: region, disability status, family history of heart disease or stroke, alcohol consumption, physical activity, body mass index (BMI), hypertension, diabetes, dyslipidemia, and the Charlson Comorbidity Index (CCI). Questionnaire- and examination-based covariates were obtained from the most recent health screening before the index date. BMI was classified following Asia-Pacific criteria [16]: underweight (<18.5 kg/m^2^), normal weight (18.5–22.9 kg/m^2^), overweight (23.0–24.9 kg/m^2^), and obesity (≥25.0 kg/m^2^). Hypertension, diabetes, and dyslipidemia were identified using screening results suggestive of disease (Supplementary Material 1, Supplementary Table S1), documented disease history, or use of medication for the relevant condition.

### Statistical analysis

Exact matching was applied to improve the comparability between cases and controls and minimize confounding by factors associated with both smoking behavior and disease occurrence, in accordance with the established methodological principles for case-control studies [17–19]. The standardized mean differences were measured to assess balance on matching variables, and all were below 0.10. The cases and controls were matched on sex, age, and insurance premium quantile, with up to six controls randomly selected for each case within the same matching stratum. Controls were drawn from participants with no record of cardiovascular or cerebrovascular disease during follow-up, consistent with a cumulative sampling design [19]. Because smoking trajectories were defined using repeated preindex assessments and disease status was determined after follow-up, the study design helped preserve temporal ordering and reduce the likelihood of reverse causation.

Smoking trajectories during the 6 years preceding the index date were identified using group-based trajectory modeling, which is a semiparametric approach for identifying distinct longitudinal patterns within a population [7, 8]. In the trajectory analysis, time was treated as the longitudinal scale and smoking behavior as the repeated outcome variable. Because smoking behavior was measured as an ordered quantitative variable with numeric intervals, trajectories were estimated using the censored normal (CNORM) model for continuous outcomes, as described by Jones and Nagin [20]. To allow for changes in smoking behaviors over time, polynomials up to the cubic order were assessed, and the appropriate order for each trajectory group was determined during model fitting. The candidate solutions with two to five groups using the Akaike information criterion (AIC) and Bayesian information criterion (BIC) were compared. Solutions with six or more groups could not be estimated because of nonconvergence and were excluded from model selection.

The associations between smoking trajectory groups and cardiovascular and cerebrovascular diseases were estimated using conditional logistic regression to obtain odds ratios (ORs) and 95% confidence intervals (CIs) within matched strata [21, 22]. The matched sets were treated as strata in the regression models, and the matching variables (sex, age, and insurance premium quantile) were not additionally included as covariates. Model 1 included only smoking trajectory groups. Model 2 additionally adjusted for region, family history, and disability status. Model 3 was further adjusted for alcohol consumption, physical activity, and BMI. Model 4 was additionally adjusted for hypertension, diabetes, and dyslipidemia. In Model 5, the individual metabolic conditions in Model 4 were replaced with the CCI to account for the overall comorbidity burden.

The robustness of the primary findings was assessed via sensitivity analyses. First, conditional logistic regression analyses were repeated separately among those participants whose most recent health screening was conducted within 1 year before the index date and among those screened between 1 and 2 years before the index date. Second, to assess the validity of the trajectory-based approach, we compared the model fits between the trajectory-based smoking measures and single time-point smoking measures that were assessed 1 and 6 years before the index date using the AIC and BIC. All analyses were conducted using SAS version 9.4 (SAS Institute Inc., Cary, NC, USA), and trajectory modeling was performed using the PROC TRAJ procedure [23]. Statistical significance was set at a two-sided P value of <0.05.

## Results

### Baseline characteristics

Compared with the controls, the cases were more likely to reside in small-to-medium-sized cities or rural areas and to have a family history of heart disease or stroke and a disability status (Supplementary Material 1, Supplementary Table S2). The cases also showed less favorable lifestyle profiles, including heavier smoking, higher alcohol consumption, physical inactivity, and obesity, as well as a higher prevalence of metabolic conditions and a greater overall comorbidity burden, as reflected by the higher CCI scores.

### Smoking trajectory groups

The smoking trajectory groups were identified over the 6 years preceding the index date (Figure 1; Supplementary Material 1, Supplementary Table S3). Model fit was assessed for models with two to five groups using the AIC and BIC. The adequacy of group assignment was assessed using the average posterior probability of group membership, with values ≥0.70 considered acceptable [24, 25]. A five-group model was selected as the final model because it showed the best fit according to the AIC and BIC values and met the minimum group size criterion of 5% for all groups [25]. The final model identified five trajectory groups: never (50.6%), former (23.6%), high-decreasing (5.2%), moderate-stable (12.4%), and high-stable (8.2%) groups.

**FIGURE 1:**
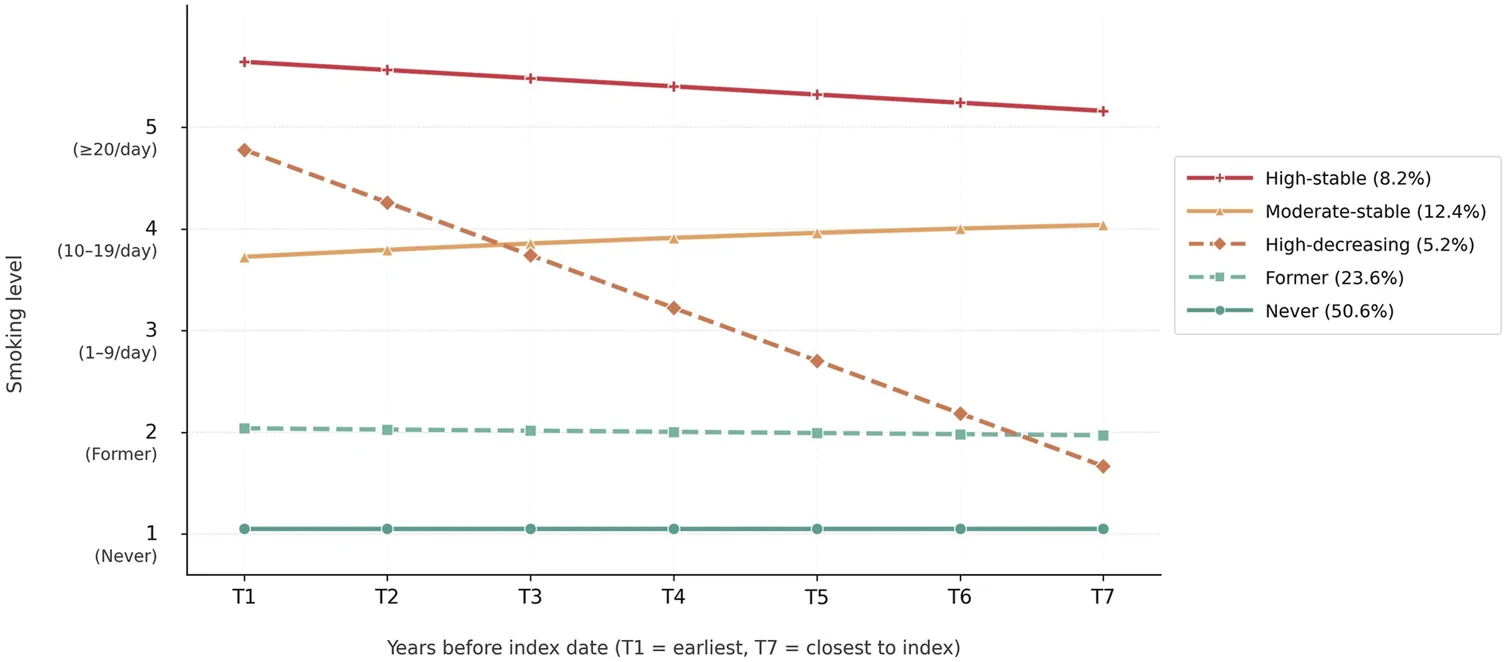
Smoking trajectory groups during the 6-year prediagnostic period, South Korea, 2011–2022. Note: The final model identified five smoking trajectory groups. T7 denotes the smoking assessment closest to the index date. Predicted trajectory values were estimated using a continuous approximation and may slightly exceed the categorical range of the smoking variable, from 1 to 5.

The never group was the largest and comprised higher proportions of women, individuals in their 60s, lower-income individuals, and rural residents (Table 1). The former group comprised higher proportions of men, older adults, and higher-income individuals and showed the highest proportion of family history of heart disease or stroke. The high-decreasing and moderate-stable groups were predominantly male and included relatively high proportions of individuals in their 40s and 50s. The high-decreasing group was additionally characterized by a higher prevalence of obesity, whereas the moderate-stable group showed more frequent alcohol consumption. The high-stable group was characterized by higher proportions of men, individuals in their 50s, and lower-income individuals and showed the highest prevalence of rural residence, disability, frequent alcohol consumption, physical inactivity, and metabolic conditions.

**TABLE 1:** Descriptive characteristics by the smoking trajectory groups, South Korea, 2011–2022.

Category	Group 1 (Never)		Group 2 (Former)		Group 3 (High-decreasing)		Group 4 (Moderate-stable)		Group 5 (High-stable)	
	N	%	N	%	N	%	N	%	N	%
All*	559,803	50.6	261,279	23.6	57,294	5.2	137,453	12.4	90,399	8.2
Sex										
Male	182,317	32.6	251,691	96.3	54,836	95.7	132,047	96.1	89,423	98.9
Female	377,486	67.4	9,588	3.7	2,458	4.3	5,406	3.9	976	1.1
Age group										
30–39	13,996	2.5	6,142	2.4	2,723	4.8	8,667	6.3	2,902	3.2
40–49	73,290	13.1	39,922	15.3	12,416	21.7	35,657	25.9	20,089	22.2
50–59	205,319	36.7	100,637	38.5	22,419	39.1	53,985	39.3	39,699	43.9
60–69	267,198	47.7	114,578	43.9	19,736	34.4	39,144	28.5	27,709	30.7
Income quantiles (Q1–Q5)										
Q1 (lowest)	108,655	19.4	32,834	12.6	7,384	12.9	17,120	12.5	13,379	14.8
Q2	91,058	16.3	34,276	13.1	7,458	13.0	16,717	12.2	11,654	12.9
Q3	98,306	17.6	44,177	16.9	10,210	17.8	25,277	18.4	17,224	19.1
Q4	112,049	20.0	58,113	22.2	13,780	24.1	34,884	25.4	22,801	25.2
Q5 (highest)	149,735	26.7	91,879	35.2	18,462	32.2	43,455	31.6	25,341	28.0
Region										
Metropolitan area	241,131	43.1	114,428	43.8	24,248	42.3	58,534	42.6	36,785	40.7
Urban area	269,625	48.2	129,656	49.6	28,808	50.3	69,693	50.7	45,300	50.1
Rural area	49,047	8.8	17,195	6.6	4,238	7.4	9,226	6.7	8,314	9.2
Family history of heart disease or stroke										
No	484,322	86.5	221,868	84.9	50,009	87.3	120,281	87.5	78,501	86.8
Yes	75,481	13.5	39,411	15.1	7,285	12.7	17,172	12.5	11,898	13.2
Disability										
No	532,268	95.1	245,309	93.9	53,754	93.8	129,629	94.3	84,562	93.5
Yes	27,535	4.9	15,970	6.1	3,540	6.2	7,824	5.7	5,837	6.5
Smoking										
Never	543,532	97.1	34,458	13.2	11,103	19.4	367	0.3	99	0.1
Former	16,046	2.9	209,455	80.2	37,549	65.5	4,778	3.5	1,185	1.3
1–9 cigarettes per day	180	0.0	9,972	3.8	5,952	10.4	14,260	10.4	649	0.7
10–19 cigarettes per day	45	0.0	6,068	2.3	2,458	4.3	93,904	68.3	14,031	15.5
≥20 cigarettes per day	0	0.0	1,326	0.5	232	0.4	24,144	17.6	74,435	82.3
Alcohol consumption										
Non-drinker	321,345	57.4	61,398	23.5	14,341	25.0	25,939	18.9	19,944	22.1
1–2 times per week	207,041	37.0	144,054	55.1	29,743	51.9	77,038	56.0	39,889	44.1
3–4 times per week	23,832	4.3	40,218	15.4	9,308	16.2	25,499	18.6	19,625	21.7
≥5 times per week	7,585	1.4	15,609	6.0	3,902	6.8	8,977	6.5	10,941	12.1
Physical activity										
Inactive	191,917	34.3	64,616	24.7	18,553	32.4	45,333	33.0	38,474	42.6
1–2 times per week	285,522	51.0	149,357	57.2	30,135	52.6	73,976	53.8	42,222	46.7
3–4 times per week	54,111	9.7	31,775	12.2	5,721	10.0	12,280	8.9	6,190	6.8
≥5 times per week	28,253	5.0	15,531	5.9	2,885	5.0	5,864	4.3	3,513	3.9
BMI										
Underweight (<18.5)	10,554	1.9	2,086	0.8	651	1.1	2,649	1.9	1,567	1.7
Normal (18.5–22.9)	193,423	34.6	60,134	23.0	13,441	23.5	40,425	29.4	25,539	28.3
Overweight (23.0–24.9)	147,520	26.4	75,458	28.9	15,293	26.7	36,480	26.5	23,235	25.7
Obesity (≥25.0)	208,306	37.2	123,601	47.3	27,909	48.7	57,899	42.1	40,058	44.3
CCI										
0	228,965	40.9	111,571	42.7	24,810	43.3	66,436	48.3	38,980	43.1
1	158,080	28.2	71,945	27.5	15,377	26.8	35,588	25.9	23,837	26.4
2	84,690	15.1	37,823	14.5	7,916	13.8	16,980	12.4	12,604	13.9
≥3	88,068	15.7	39,940	15.3	9,191	16.0	18,449	13.4	14,978	16.6

* Overall percentages indicate between-group proportions, whereas category-specific percentages for each variable indicate within-group proportions.

### Main associations between smoking trajectories and cardiovascular and cerebrovascular diseases

Table 2 shows the conditional logistic regression results for the associations between smoking trajectories and cardiovascular and cerebrovascular diseases. The direction and magnitude of the associations were largely similar across models. Model 5, adjusted for sociodemographic factors, health-related factors, and the CCI, was used as the primary fully adjusted model because it accounted for the overall comorbidity burden using the CCI.

**TABLE 2:** Associations between smoking trajectory groups and cardiovascular and cerebrovascular disease, South Korea, 2011–2022.

Variable	OR (95% CI)				
	M1^a^	M2^b^	M3^c^	M4^d^	M5^e^
Trajectory groups					
Never (ref.)	​	​	​	​	​
Former	1.13 (1.12–1.15)^***^	1.13 (1.11–1.14)^***^	1.14 (1.12–1.16)^***^	1.13 (1.11–1.15)^***^	1.11 (1.09–1.13)^***^
High-decreasing	1.43 (1.40–1.46)^***^	1.43 (1.40–1.46)^***^	1.43 (1.40–1.47)^***^	1.41 (1.38–1.44)^***^	1.36 (1.33–1.40)^***^
Moderate-stable	1.57 (1.54–1.60)^***^	1.57 (1.55–1.60)^***^	1.62 (1.60–1.65)^***^	1.61 (1.58–1.63)^***^	1.59 (1.56–1.62)^***^
High-stable	2.05 (2.01–2.09)^***^	2.05 (2.01–2.09)^***^	2.07 (2.03–2.11)^***^	2.02 (1.98–2.06)^***^	1.97 (1.93–2.01)^***^
Region					
Metropolitan area (ref.)	​	​	​	​	​
Urban area	​	1.07 (1.06–1.08)^***^	1.06 (1.05–1.07)^***^	1.05 (1.04–1.06)^***^	1.05 (1.04–1.06)^***^
Rural area	​	1.18 (1.16–1.20)^***^	1.14 (1.12–1.16)^***^	1.13 (1.11–1.15)^***^	1.09 (1.07–1.11)^***^
Family history of heart disease or stroke					
No (ref.)	​	​	​	​	​
Yes	​	1.36 (1.34–1.38)^***^	1.37 (1.35–1.39)^***^	1.35 (1.33–1.36)^***^	1.36 (1.34–1.37)^***^
Disability					
No (ref.)	​	​	​	​	​
Yes	​	1.34 (1.31–1.36)^***^	1.31 (1.29–1.34)^***^	1.28 (1.26–1.31)^***^	1.19 (1.16–1.21)^***^
Alcohol consumption per week					
Non-drinker (ref.)	​	​	​	​	​
1–2 times	​	​	0.89 (0.88–0.90)^***^	0.89 (0.88–0.90)^***^	0.93 (0.92–0.94)^***^
3–4 times	​	​	0.88 (0.86–0.89)^***^	0.84 (0.83–0.86)^***^	0.91 (0.89–0.92)^***^
≥5 times	​	​	0.91 (0.89–0.94)^***^	0.87 (0.85–0.89)^***^	0.93 (0.91–0.95)^***^
Physical activity per week					
Inactive (ref.)	​	​	​	​	​
1–2 times	​	​	0.88 (0.87–0.89)^***^	0.89 (0.88–0.90)^***^	0.90 (0.89–0.91)^***^
3–4 times	​	​	0.84 (0.83–0.86)^***^	0.85 (0.84–0.87)^***^	0.87 (0.85–0.88)^***^
≥5 times	​	​	0.83 (0.81–0.85)^***^	0.84 (0.82–0.86)^***^	0.84 (0.82–0.86)^***^
BMI					
Normal (ref.)	​	​	​	​	​
Underweight (<18.5)	​	​	0.81 (0.77–0.84)^***^	0.84 (0.81–0.88)^***^	0.81 (0.78–0.84)^***^
Overweight (23.0–24.9)	​	​	1.20 (1.19–1.22)^***^	1.15 (1.13–1.16)^***^	1.17 (1.15–1.18)^***^
Obesity (≥25.0)	​	​	1.40 (1.38–1.41)^***^	1.24 (1.22–1.25)^***^	1.28 (1.26–1.29)^***^
Hypertension					
No (ref.)	​	​	​	​	​
Yes	​	​	​	1.44 (1.42–1.45)^***^	​
Diabetes					
No (ref.)	​	​	​	​	​
Yes	​	​	​	1.31 (1.29–1.33)^***^	​
Dyslipidemia					
No (ref.)	​	​	​	​	​
Yes	​	​	​	1.12 (1.11–1.13)^***^	​
CCI					
0 (ref.)	​	​	​	​	​
1	​	​	​	​	1.57 (1.55–1.59)^***^
2	​	​	​	​	2.28 (2.25–2.31)^***^
≥3	​	​	​	​	3.88 (3.83–3.93)^***^
AIC	705,257	701,871	696,890	688,421	655,929
BIC	705,304	701,966	697,093	688,659	656,168

* P < 0.05, **P < 0.01, ***P < 0.001. OR, odds ratio; CI, confidence interval; AIC, akaike information criterion; BIC, bayesian information criterion; BMI, body mass index; CCI, charlson comorbidity index.

^a^ M1: smoking trajectory groups only.

^b^ M2: M1 + region, family history of heart disease or stroke, and disability.

^c^ M3: M2 + alcohol consumption, physical activity, and BMI.

^d^ M4: M3 + hypertension, diabetes, and dyslipidemia.

^e^ M5: M3 + CCI.

In Model 5, compared with the never group, the odds of cardiovascular and cerebrovascular diseases were highest in the high-stable group (OR = 1.97; 95% CI 1.93–2.01), followed by the moderate-stable group (OR = 1.59; 95% CI 1.56–1.62), high-decreasing group (OR = 1.36; 95% CI 1.33–1.40), and former group (OR = 1.11; 95% CI 1.09–1.13).

### Subtype-specific associations

Table 3 shows the subtype-specific associations from Model 5. Compared with the never group, the high-stable group showed increased odds across all disease subtypes. The strongest association was observed for acute myocardial infarction (OR = 3.51; 95% CI 3.34–3.69), followed by cerebral infarction (OR = 2.46; 95% CI 2.35–2.56), ischemic heart disease (OR = 2.04; 95% CI 1.99–2.09), overall cerebrovascular disease (OR = 1.90; 95% CI 1.84–1.96), and intracranial hemorrhage (OR = 1.49; 95% CI 1.37–1.61). All these associations were statistically significant (P < 0.001), with larger estimates for ischemic outcomes than for intracranial hemorrhage.

**TABLE 3:** Associations between smoking trajectory groups and cardiovascular and cerebrovascular disease subtypes, South Korea, 2011–2022.

Variable	OR (95% CI)					
	D0^a^	D1^b^	D2^c^	D3^d^	D4^e^	D5^f^
Trajectory groups						
Never (ref.)	​	​	​	​	​	​
Former	1.11 (1.09–1.13)***	1.17 (1.14–1.19)***	1.13 (1.08–1.18)***	1.03 (1.01–1.05) *	0.95 (0.89–1.02)	0.99 (0.96–1.03)
High-decreasing	1.36 (1.33–1.40)***	1.43 (1.38–1.47)***	1.62 (1.52–1.73)***	1.28 (1.24–1.33)***	1.10 (1.00–1.22)	1.37 (1.30–1.45)***
Moderate-stable	1.59 (1.56–1.62)***	1.59 (1.55–1.63)***	2.55 (2.43–2.68)***	1.60 (1.56–1.64)***	1.34 (1.25–1.43)***	1.98 (1.90–2.05)***
High-stable	1.97 (1.93–2.01)***	2.04 (1.99–2.09)***	3.51 (3.34–3.69)***	1.90 (1.84–1.96)***	1.49 (1.37–1.61)***	2.46 (2.35–2.56)***
Region						
Metropolitan area (ref.)	​	​	​	​	​	​
Urban area	1.05 (1.04–1.06)***	1.00 (0.99–1.02)	1.01 (0.98–1.04)	1.11 (1.10–1.13)***	1.00 (0.96–1.04)	1.05 (1.02–1.07)***
Rural area	1.09 (1.07–1.11)***	1.00 (0.98–1.03)	1.03 (0.97–1.09)	1.20 (1.17–1.23)***	1.13 (1.05–1.21)***	1.19 (1.14–1.24)***
Family history of heart disease or stroke						
No (ref.)	​	​	​	​	​	​
Yes	1.36 (1.34–1.37)***	1.47 (1.44–1.50)***	1.41 (1.35–1.47)***	1.24 (1.22–1.27)***	1.14 (1.08–1.21)***	1.20 (1.16–1.24)***
Disability						
No (ref.)	​	​	​	​	​	​
Yes	1.19 (1.16–1.21)***	1.17 (1.14–1.21)***	1.04 (0.98–1.11)	1.21 (1.17–1.24)***	1.49 (1.37–1.61)***	1.23 (1.17–1.28)***
Alcohol consumption per week						
Non-drinker (ref.)	​	​	​	​	​	​
1–2 times	0.93 (0.92–0.94)***	0.86 (0.85–0.88)***	0.66 (0.64–0.69)***	1.00 (0.99–1.02)	1.03 (0.99–1.08)	0.96 (0.93–0.99)**
3–4 times	0.91 (0.89–0.92)***	0.79 (0.77–0.81)***	0.46 (0.44–0.49)***	1.07 (1.04–1.10)***	1.30 (1.21–1.39)***	1.01 (0.97–1.05)
≥5 times	0.93 (0.91–0.95)***	0.77 (0.74–0.80)***	0.41 (0.38–0.44)***	1.17 (1.13–1.21)***	1.61 (1.46–1.76)***	1.04 (0.99–1.10)
Physical activity per week						
Inactive (ref.)	​	​	​	​	​	​
1–2 times	0.90 (0.89–0.91)***	0.93 (0.91–0.94)***	0.94 (0.91–0.97)***	0.88 (0.87–0.89)***	0.89 (0.85–0.93)***	0.84 (0.82–0.86)***
3–4 times	0.87 (0.85–0.88)***	0.87 (0.85–0.89)***	0.89 (0.84–0.94)***	0.86 (0.84–0.89)***	0.86 (0.81–0.93)***	0.83 (0.80–0.87)***
≥5 times	0.84 (0.82–0.86)***	0.87 (0.85–0.90)***	0.93 (0.87–1.00) *	0.81 (0.78–0.83)***	0.87 (0.80–0.96)**	0.76 (0.72–0.80)***
BMI						
Normal (ref.)	​	​	​	​	​	​
Underweight (<18.5)	0.81 (0.78–0.84)***	0.70 (0.66–0.75)***	0.64 (0.54–0.75)***	0.89 (0.84–0.94)***	1.13 (0.99–1.29)	0.78 (0.70–0.86)***
Overweight (23.0–24.9)	1.17 (1.15–1.18)***	1.23 (1.21–1.26)***	1.30 (1.24–1.35)***	1.11 (1.09–1.13)***	1.07 (1.02–1.13)**	1.18 (1.15–1.22)***
Obesity (≥25.0)	1.28 (1.26–1.29)***	1.43 (1.41–1.46)***	1.55 (1.49–1.61)***	1.13 (1.11–1.15)***	1.09 (1.04–1.14)***	1.28 (1.24–1.31)***
CCI						
0 (ref.)	​	​	​	​	​	​
1	1.57 (1.55–1.59)***	1.52 (1.50–1.55)***	1.37 (1.32–1.42)***	1.62 (1.59–1.65)***	1.23 (1.18–1.29)***	1.31 (1.27–1.35)***
2	2.28 (2.25–2.31)***	2.16 (2.12–2.20)***	1.93 (1.84–2.01)***	2.42 (2.37–2.47)***	1.63 (1.54–1.73)***	1.77 (1.71–1.83)***
≥3	3.88 (3.83–3.93)***	3.56 (3.49–3.63)***	3.61 (3.46–3.76)***	4.27 (4.19–4.35)***	3.64 (3.45–3.84)***	3.74 (3.63–3.86)***

* P < 0.05, **P < 0.01, ***P < 0.001. OR, odds ratio; CI, confidence interval; BMI, body mass index; CCI, charlson comorbidity index.

Results are from Model 5, adjusted for region, family history of heart disease or stroke, disability, alcohol consumption, physical activity, body mass index, and Charlson Comorbidity Index.

^a^ D0: cardiovascular and cerebrovascular disease (ICD-10, codes I20–I25 and I60–I69).

^b^ D1: ischemic heart disease (ICD-10, codes I20–I25).

^c^ D2: acute myocardial infarction (ICD-10, code I21).

^d^ D3: cerebrovascular disease (ICD-10, codes I60–I69).

^e^ D4: intracranial hemorrhage (ICD-10, codes I60–I62).

^f^ D5: cerebral infarction (ICD-10, code I63).

### Sensitivity analyses

Supplementary Tables S4, S5 (Supplementary Material 1) show the results of the sensitivity analyses. In the analyses stratified by the timing of the most recent health screening before the index date, the pattern of associations across the smoking trajectory groups was similar to that observed in the main analysis. Moreover, the trajectory-based smoking measures showed a better model fit than the single time-point smoking measures at both time points, as indicated by the lower AIC and BIC values.

## Discussion

Using longitudinal smoking data from the Korean national health screening program, this study identified distinct prediagnostic smoking trajectories and examined their associations with cardiovascular and cerebrovascular disease risk. The odds of disease increased with greater smoking intensity and persistence, with the highest odds observed in the high-stable group and lower odds in the high-decreasing group than in the moderate-stable and high-stable groups. Associations were particularly pronounced for ischemic outcomes, including acute myocardial infarction and cerebral infarction. These patterns suggest that trajectories defined in the years immediately before diagnosis may better capture recent changes and persistence in smoking exposure than trajectories defined from cohort enrollment.

The increased risk observed in the high-stable group is consistent with the findings of previous studies using repeated smoking measures or trajectory-based approaches. Jee et al. reported that the high-stable group had significantly higher cardiovascular and cerebrovascular disease hazards than the never group (HR = 1.46) [9]. Damigou et al., in a lifestyle trajectory study incorporating smoking behavior, reported that continuous smoking was associated with higher cardiovascular disease risk than never smoking (HR = 2.77) [10]. Rissanen et al. similarly classified the smoking trajectories according to cessation timing and found that persistent smokers showed the highest hazard of stroke compared with never smokers (HR = 1.69) [11]. Across these methodologically diverse studies, the evidence consistently points to an association between sustained smoking exposure and increased cardiovascular and cerebrovascular risk.

However, the ordering of subtype-specific associations in the high-stable group differed from that reported in a previous Korean trajectory study. In our subtype analysis, the high-stable group showed the strongest association with acute myocardial infarction (OR = 3.51). Among the subtypes examined in both studies, cerebral infarction showed the strongest association in the present study, whereas ischemic heart disease showed the strongest association in Jee et al. [9]. These differences may partly reflect variations in the study design, population characteristics, outcome definition, and analytic framework.

The high-decreasing group consistently showed lower odds than both the moderate-stable and high-stable groups across the disease subtypes, particularly for ischemic outcomes. A previous trajectory-based study similarly reported lower hazards of ischemic heart disease among individuals with decreasing smoking intensity than among those with persistently high smoking intensity, a pattern that is consistent with the findings of the present study [9]. Although complete cessation remains the most effective preventive strategy, these findings suggest that reducing smoking intensity may still be associated with lower odds of cardiovascular and cerebrovascular diseases than continued heavy smoking [26, 27]. The stronger associations observed for the ischemic outcomes are also consistent with previous trajectory-based studies [9–11]. These patterns are biologically plausible, considering the known effects of smoking on endothelial dysfunction, thrombosis, oxidative stress, and atherosclerotic progression, which are processes that are more closely associated with ischemic events than hemorrhagic vascular events [28, 29].

Moreover, the former group showed higher odds of heart disease outcomes than the never group, whereas no substantial elevation was observed for the cerebrovascular outcomes, suggesting that the time course of risk reduction may differ across vascular disease subtypes. Coronary risk may remain elevated for a prolonged period after cessation, particularly among those with heavier previous smoking exposure [30, 31], whereas stroke risk appears to decline more rapidly after quitting [32]. Our findings reinforce the importance of cessation for maximizing long-term cardiovascular benefit.

The sensitivity analyses supported the robustness of the primary findings. Associations were consistent across subgroups defined by the timing of the most recent health screening, and the trajectory-based smoking measures showed better model fit than the single time-point measures, suggesting that repeated assessments may better capture the aspects of cumulative smoking exposure not reflected by a single measure.

The present study has several strengths. First, trajectory modeling was applied to repeated preindex smoking assessments, identifying distinct longitudinal patterns of smoking intensity and persistence in the period immediately preceding the index date. This approach extends previous evidence based mainly on baseline smoking status by showing that prediagnostic smoking patterns are associated with subtype-specific cardiovascular and cerebrovascular disease risk. Second, by defining the smoking trajectories over the 6 years preceding the index date, the study preserved the temporal ordering between exposure and outcomes and reduced the likelihood of reverse causation, a limitation observed in studies relying on a single baseline smoking assessment obtained at cohort entry. Third, the use of a large nationwide matched case-control design based on health insurance data improved the population coverage and comparability between cases and controls.

### Limitations

Several limitations should be noted. First, the study population was restricted to national health screening participants, which may introduce healthy screenee bias and limit the generalizability of the findings to nonparticipants. Second, smoking status was based on self-reported questionnaire data and may be subject to measurement error or reporting bias. Age at smoking initiation is an important aspect of smoking history, but this information was not collected in the health screening questionnaire and could not be assessed. Residual confounding by unmeasured factors, including education, employment status, deprivation, diet, sleep, and more detailed measures of alcohol consumption and physical activity, also cannot be excluded. Third, reverse causality remains possible because some participants may have reduced or stopped smoking in response to preclinical or existing health conditions before the index date. Although the consistency of associations across subgroups defined by the timing of the most recent screening supports the robustness of the findings, reverse causality cannot be fully excluded. Fourth, although classification diagnostics supported the adequacy of the selected trajectory model, group assignment in trajectory modeling is probabilistic and may be influenced by model-selection decisions. Finally, the 6-year observation window may not have fully captured the cumulative vascular effects of longer-term smoking exposure, as cardiovascular risks associated with smoking can persist over decades [33, 34]. Future studies with longer prediagnostic exposure histories are needed to determine whether prolonged smoking patterns confer additional vascular risk.

### Conclusions

Smoking trajectories over the 6 years preceding the index date were associated with cardiovascular and cerebrovascular disease, with progressively higher odds in groups characterized by greater smoking intensity and persistence. Associations were stronger for ischemic than for hemorrhagic outcomes. Repeated smoking information from population-based screening programs may characterize prediagnostic exposure patterns and identify sustained high-risk smoking profiles for earlier cessation intervention.
