# Supplementary material for: Prediagnostic smoking intensity trajectories and subtype-specific vascular disease risk in a nationwide health screening population
Source: Int J Public Health. 2026 Oct 29;71:1610024. doi: 10.3389/ijph.2026.1610024 (PMC13754752; doi:10.3389/ijph.2026.1610024)
Supplement: Supplementary file 1 [file Supplementaryfile1.docx]

**Supplementary File 1**

***Journal: International Journal of Public Health***

**Title: Prediagnostic smoking intensity trajectories and subtype-specific vascular disease risk in a nationwide health screening population**

**Figure S1.** Construction of the matched study population for the smoking trajectory analysis, South Korea, 2011–2022 (p.2)

**Table S1.** Classification criteria for the test results in the Korean national health screening program, South Korea (p.3)

**Table S2.** Descriptive characteristics of cases and controls in the final matched study population, South Korea, 2011–2022 (p.4)

**Table S3.** Model selection results for group-based trajectory modeling, South Korea, 2011–2022 (p.5)

**Table S4.** Associations between smoking trajectory groups and cardiovascular and cerebrovascular disease stratified by timing of the most recent health screening, South Korea, 2011–2022 (p.6)

**Table S5.** Comparison of trajectory-based and single time-point smoking measures: odds ratios and model fit, South Korea, 2011–2022 (p.7)

**Note:** Preindex eligibility criteria were applied to cases before matching. Potential controls were exactly matched to these cases by sex, age, and insurance premium quantile, with up to six controls selected for each retained case. Matched controls were assigned the index date of the corresponding case, after which the same preindex eligibility criteria were applied. Complete matched sets were included in the final analysis.

Figure S1. Construction of the matched study population for the smoking trajectory analysis, South Korea, 2011–2022

Table S1. Classification criteria for the test results in the Korean national health screening program, South Korea.

| Target disease | Test Item | Test result | | |
| --- | --- | --- | --- | --- |
|  |  | Normal (A) | Normal (B) | Suspected |
| Hypertension | Systolic blood pressure | $<$120 | 120–139 | $\geq$ 140 |
|  | Diastolic blood pressure (mmHg) | $<$80 | 80–89 | $\geq$ 90 |
| Diabetes | Fasting blood glucose (mg/dL) | $<$100 | 100–125 | $\geq$ 126 |
| Dyslipidemia | Total cholesterol (mg/dL) | $<$200 | 200–239 | $\geq$ 240 |
|  | HDL cholesterol (mg/dL) | $\geq$60 | 40–59 | $<$40 |
|  | Triglycerides (mg/dL) | $<$150 | 150–199 | $\geq$ 200 |
|  | LDL cholesterol (mg/dL) | $<$130 | 130–159 | $\geq$ 160 |

**HDL, high-density lipoprotein; LDL, low-density lipoprotein. Normal (B) denotes a borderline range in the Korean national health screening classification.**

**Table S2.** Descriptive characteristics of cases and controls in the final matched study population, South Korea, 2011–2022.

| Variables | Case | |  | Control | |
| --- | --- | --- | --- | --- | --- |
|  | N | % |  | N | % |
| All | 241,163 | 100 |  | 865,065 | 100 |
| Sex |  |  |  |  |  |
| Male | 151,263 | 62.7 |  | 559,051 | 64.6 |
| Female | 89,900 | 37.3 |  | 306,014 | 35.4 |
| Age group |  |  |  |  |  |
| Age, mean ± SD | 59.4 ± 7.6 | |  | 58.8 ± 7.6 | |
| 30–39 | 6,710 | 2.8 |  | 27,720 | 3.2 |
| 40–49 | 35,470 | 14.7 |  | 145,904 | 16.9 |
| 50–59 | 86,488 | 35.9 |  | 335,571 | 38.8 |
| 60–69 | 112,495 | 46.6 |  | 355,870 | 41.1 |
| Income quantile (Q1–Q5) |  |  |  |  |  |
| Q1(lowest) | 40,602 | 16.8 |  | 138,770 | 16.0 |
| Q2 | 35,507 | 14.7 |  | 125,656 | 14.5 |
| Q3 | 42,814 | 17.8 |  | 152,380 | 17.6 |
| Q4 | 52,939 | 22.0 |  | 188,688 | 21.8 |
| Q5 (highest) | 69,301 | 28.7 |  | 259,571 | 30.0 |
| Region |  |  |  |  |  |
| Metropolitan area | 100,126 | 41.5 |  | 375,000 | 43.3 |
| Urban area | 119,524 | 49.6 |  | 423,558 | 49.0 |
| Rural area | 21,513 | 8.9 |  | 66,507 | 7.7 |
| Family history of heart disease or stroke |  |  |  |  |  |
| No | 201,341 | 83.5 |  | 753,640 | 87.1 |
| Yes | 39,822 | 16.5 |  | 111,425 | 12.9 |
| Disability |  |  |  |  |  |
| No | 224,413 | 93.1 |  | 821,109 | 94.9 |
| Yes | 16,750 | 6.9 |  | 43,956 | 5.1 |
| Smoking per day |  |  |  |  |  |
| Never | 123,690 | 51.3 |  | 465,869 | 53.9 |
| Former | 53,289 | 22.1 |  | 215,724 | 24.9 |
| 1–9 cigarettes | 6,898 | 2.9 |  | 24,115 | 2.8 |
| 10–19 cigarettes | 27,801 | 11.5 |  | 88,705 | 10.3 |
| ≥ 20 cigarettes | 29,485 | 12.2 |  | 70,652 | 8.2 |
| Alcohol consumption per week |  |  |  |  |  |
| Non-drinker | 102,512 | 42.5 |  | 340,455 | 39.4 |
| 1–2 times | 102,105 | 42.3 |  | 395,660 | 45.7 |
| 3–4 times | 25,455 | 10.6 |  | 93,027 | 10.8 |
| ≥ 5 times | 11,091 | 4.6 |  | 35,923 | 4.2 |
| Physical activity per week |  |  |  |  |  |
| Inactive | 86,817 | 36.0 |  | 272,076 | 31.5 |
| 1–2 times | 121,508 | 50.4 |  | 459,704 | 53.1 |
| 3–4 times | 21,697 | 9.0 |  | 88,380 | 10.2 |
| ≥ 5 times | 11,141 | 4.6 |  | 44,905 | 5.2 |
| BMI |  |  |  |  |  |
| Underweight (<18.5) | 2,987 | 1.2 |  | 14,520 | 1.7 |
| Normal (18.5–22.9) | 63,951 | 26.5 |  | 269,011 | 31.1 |
| Overweight (23.0–24.9) | 64,126 | 26.6 |  | 233,860 | 27.0 |
| Obesity (≥ 25.0) | 110,099 | 45.7 |  | 347,674 | 40.2 |
| CCI |  |  |  |  |  |
| 0 | 66,492 | 27.6 |  | 404,270 | 46.7 |
| 1 | 62,806 | 26.0 |  | 242,021 | 28.0 |
| 2 | 44,086 | 18.3 |  | 115,927 | 13.4 |
| ≥ 3 | 67,779 | 28.1 |  | 102,847 | 11.9 |

**BMI, body mass index; CCI, Charlson Comorbidity Index; SD, standard deviation.
The sociodemographic and eligibility variables were defined at the end of the most recent screening year before the index date, whereas the health examination-based variables were based on the most recent health examination before the index date.**

Table S3. Model selection results for group-based trajectory modeling, South Korea, 2011–2022.

| Number of groups  (order by group) | BIC | AIC | Group membership proportion (%) | | | | |
| --- | --- | --- | --- | --- | --- | --- | --- |
|  |  |  | Group 1 | Group 2 | Group 3 | Group 4 | Group 5 |
| 2 (01) | −5,866,081 | −5,866,051 | 75.4 | 24.6 | - | - | - |
| 3 (012) | −5,559,942 | −5,559,888 | 56.1 | 23.2 | 20.8 | - | - |
| 4 (0112) | −5,223,730 | −5,223,659 | 54.2 | 23.4 | 5.2 | 17.3 | - |
| 5 (01121) | −4,940,507 | −4,940,418 | 50.6 | 23.6 | 5.2 | 12.4 | 8.2 |

AIC, Akaike information criterion; BIC, Bayesian information criterion.
Smoking (1–5 scale): 1, never; 2, former; 3, 1–9 cigarettes per day; 4, 10–19 cigarettes per day; 5, ≥ 20 cigarettes per day.
Values in parentheses indicate the polynomial order specified for each trajectory group.

**Table S4.** Associations between smoking trajectory groups and cardiovascular and cerebrovascular disease stratified by timing of the most recent health screening, South Korea, 2011–2022.

| Trajectory groups | Screening within 1 year  (N = 748,698) | | Screening within 1–2 years  (N = 357,530) | | |
| --- | --- | --- | --- | --- | --- |
|  | OR (95% CI) | P value | OR (95% CI) | | P value |
| Never (ref.) |  |  |  | |  |
| Former | 1.11 (1.09–1.13) | <0.001 | 1.10 (1.06–1.14) | | <0.001 |
| High-decreasing | 1.39 (1.34–1.43) | <0.001 | 1.37 (1.29–1.45) | | <0.001 |
| Moderate-stable | 1.59 (1.55–1.63) | <0.001 | 1.56 (1.49–1.62) | | <0.001 |
| High-stable | 1.98 (1.93–2.03) | <0.001 | 1.99 (1.90–2.09) | | <0.001 |
| AIC | 333,394 | | | 103,027 | |
| BIC | 333,625 | | | 103,243 | |

OR, odds ratio; CI, confidence interval; AIC, Akaike information criterion; BIC, Bayesian information criterion.
Adjusted for region, family history of heart disease or stroke, disability, alcohol consumption, physical activity, body mass index, and Charlson Comorbidity Index. Screening within 1 year: cases (163,082) and controls (585,616); Screening within 1–2 years: cases (78,081) and controls (279,449).

**Table S5.** Comparison of trajectory-based and single time-point smoking measures: odds ratios and model fit, South Korea, 2011–2022.

| Trajectory groups | Trajectory-based | | Smoking status categories | Single time-point | |
| --- | --- | --- | --- | --- | --- |
|  | 1 year | 6 years |  | 1 year | 6 years |
| Never (ref.) |  |  | Never (ref.) |  |  |
| Former | 1.12^***^ | 1.12^***^ | Former | 1.09^***^ | 1.09^***^ |
| High-decreasing | 1.39^***^ | 1.47^***^ | 1–9 cigarettes per day | 1.35^***^ | 1.29^***^ |
| Moderate-stable | 1.60^***^ | 1.62^***^ | 10–19 cigarettes per day | 1.53^***^ | 1.47^***^ |
| High-stable | 2.02^***^ | 2.03^***^ | $\text{ ≥ }$20 cigarettes per day | 1.92^***^ | 1.79^***^ |
| AIC | 374,312 | 162,733 | AIC | 374,599 | 163,111 |
| BIC | 374,544 | 162,953 | BIC | 374,830 | 163,331 |

*P < 0.05, **P < 0.01, ***P < 0.001. OR, odds ratio; AIC, Akaike information criterion; BIC, Bayesian information criterion.

Adjusted for region, family history of heart disease or stroke, disability, alcohol consumption, physical activity, body mass index, and Charlson Comorbidity Index. 1-year period before the index date (N = 769,623, cases = 161,647, controls = 607,976), and 6-year period before the index date (N = 436,128, cases = 91,510, controls = 344,618).
